# Supplementary material for: The airborne transmission of viruses causes tight transmission bottlenecks
Source: Nat Commun. 2024 Apr 26;15:3540. doi: 10.1038/s41467-024-47923-z (PMC11053022; doi:10.1038/s41467-024-47923-z)
Supplement: Supplementary file 1 — Supplementary Information [file 41467_2024_47923_MOESM1_ESM.pdf]

The airborne transmission of viruses causes tight transmission bottlenecks

Supplementary Information

Index

Supplementary Methods 1. Details of the derivation of R_{env} 1

Supplementary Methods 2. Calculation of a default effective viral load 4

Supplementary Methods 3. Modelling the emission of particles 7

Supplementary Methods 4. Analytical solution of the diffusion equation 10

Supplementary Note 1. Modelling variance in transmission using a gamma-Poisson model . 13

Supplementary Note 2. Alterations and elaborations of our transmission model 13

Supplementary Note 3. Modelling variability in infectivity levels 15

Supplementary Note 4. On the removal of viruses from the air by inhalation 17

Supplementary Figures 19

Supplementary Methods 1. Details of the derivation of R_{env}

In our default model, we derived a value R_{env}=0.067 for the office environment. This value was calculated based upon the epidemiological parameter R_0, as follows.

1.1 Specification of R_{env}

For a given environment we define the equation

$$R_{\text{env}} = T_{\text{env}} \phi_{\text{env}} R_0 \tag{1}$$

where R_0 is the epidemiological parameter, T_{env} is the proportion of the infectious period that we model in our definition of an environment, and ϕ_{env} is a parameter describing the relative propensity of an environment to host cases of infection. For example if T_{env} = 1 and ϕ_{env} = 1, we would be considering the entire period of an infection in a typical environment for transmission, giving $R_{env} = R_0$. An environment in which transmission was more likely, for example containing a greater number of people, or a lower rate of ventilation, would have ϕ_{env} > 1.

1.2 Estimation of T_{env}

A previous paper describing the epidemiology of the wild-type SARS-CoV-2 strain described the infectivity distribution of this strain in terms of a gamma distribution[1].

$$I(t) = \frac{e^{-(t+s)/\beta}(t+s)^{\alpha-1}\beta^{-\alpha}}{\Gamma(\alpha)} \quad (2)$$

Where $I(t)$ is the relative infectivity at time t from the onset of symptoms, measured in units of days, and the other parameters are specified by α=8.124, β=1.572, and s=7.5. Taking the cumulative density function of this distribution, we calculated that at least 95% of infections occur within 18 days, and therefore estimated 18 days as the length of the infectious period.

In our office environment we simulate an eight-hour period of exposure to an infected individual, We make the assumption that this represents half of the waking hours of the infected individual, so that the office environment describes 1/36 of the period of infection. Specifically, for the office, T_{env} = 1/36.

1.3 Estimation of R_0

In our default model we considered a virus similar to the original strain of SARS-CoV-2. A review of the early literature identified a range of estimates for R_0, with the majority falling between 2.0 and 3.0[2]. For our calculations we used the value $R_0 = 2.5$.

1.4 Estimation of ϕ_{env}

The evaluation of the propensity of our office environment to host infections was a difficult task. The exact nature of what would be a typical environment for transmission is hard to quantify, requiring a detailed knowledge of how people who are infected with a virus, and go on to transmit that virus, move around and interact with others. We noted that SARS-CoV-2 transmission was more prevalent in household, rather than workplace environments[3]. Office workers did not have especially high mortality rates compared to other professionals during the early stages of the pandemic[4]. In the absence of further information we made the default setting $\phi_{env} = 1$ for the office environment. With the above parameter settings, this gave $R_{env} = 0.067$ for the office.

We are aware that the office environment in our model, as we describe it, does not include any short-range interactions between individuals, with the minimum distance of an uninfected person from the infected person of just under 4 metres. Our intuition is that by setting $\phi_{env} = 1$ we are overestimating the extent of transmission in the office for a realistic case of SARS-CoV-2 infection. If so, our default parameters are conservative with respect to the bottleneck sizes generated.

Supplementary Methods 2. Calculation of a default effective viral load

2.1 Calculation of effective viral load

Suppose that a person, denoted *i*, in the environment *e*, receives some physical exposure to exhaled material from an infected person. We suppose that person *i* is exposed to a mean number $P_{i,e}(r)$ particles of radius *r* μm. In our model, emitted particles had a distribution with discrete sizes, given by integer numbers *r*, and were assumed to be spherical.

Emitted particles have a specific effective viral load. We denote this as being equal to some value *L*, measured in effective viruses per ml at the time of particle emission. As such, the number of effective viruses in a single particle of radius *r* is given by

$$V(L,r) = \frac{4 * 10^{-12}}{3} L\pi r^3 \quad (3)$$

In our model a person can fail to be infected either if they are not exposed to any emitted particles, or if none of the particles to which they are exposed contain any effective virus. We can thus express the probability of a person not being infected because of exposure to particles:

$$P(\text{not infected by particles of size r}) = e^{-P_{i,e}(r)} + \sum_{d=1}^{\infty} \frac{P_{i,e}(r)^d e^{-P_{i,e}(r)}}{d!} e^{-dV(L,r)} \quad (4)$$

Or equivalently

$$P(\text{not infected by particles of size r}) = \sum_{d=0}^{\infty} \frac{P_{i,e}(r)^d e^{-P_{i,e}(r)}}{d!} e^{-dV(L,r)} \quad (5)$$

Assuming that particles act independently of one another, we next obtain the result

$$P(\text{not infected}) = \prod_r \left(\sum_{d=0}^{\infty} \frac{\left[P_{i,e}(r)\right]^d e^{-P_{i,e}(r)}}{d!} e^{-dV(L,r)} \right) \quad (6)$$

where the product is taken over all particle sizes. We now suppose that the total number of people in our environment is given by the parameter n_{env}, and assume that infections happen independently of one another. We then have an expression for R_{env}, given by

$$R_{env} = \sum_{i=1}^{n_e} \left[1 - \prod_r \left(\sum_{d=0}^{\infty} \frac{\left[P_{i,e}(r)\right]^d e^{-P_{i,e}(r)}}{d!} e^{-dV(L,r)} \right) \right] \quad (7)$$

This approach provides a conversion from a set of physical exposures and an effective viral load to the value R_{env}. Using the office as our default environment, and specifying R_{env}, we solved the equation above to derive a default value for the effective viral load $L = 3.204 \times 10^7$ effective viruses per ml.

The default effective viral load was retained across different patterns of virus emission: For example we did not rederive L given different patterns of emission e.g. from speech.

The calculations above (sections 1 and 2) affect only the default value of the effective viral load: In so far as our calculations consider a range of different values for this statistic, any error in the default value would not substantially affect our overall conclusions. However, the relationship between viral epidemiology and the size of the transmission bottleneck is important in so far as overwhelmingly high levels of exposure produce both larger transmission bottlenecks and very high numbers of infections.

2.2 Notes on the default effective viral load

Our default effective viral load is almost certainly higher than is realistic: We deduce this from two findings. Firstly, the default effective viral load is considerably greater than the published range for the number of plaque-forming units per ml for SARS-CoV-2[5]. Secondly, the probability we calculate of an individual in a high-risk environment (in our model, the lounge) is, especially for the case of continuous speech, considerably in excess of the probability derived from a recent digital study of several million person-to-person contacts[6].

The likely cause of error in our default is in our estimation of the parameter ϕ_{env}, which was set to equal 1 for the office environment. We note that the chances of transmission heavily depend upon individual environmental circumstances: While in the absence of other information, this value is a reasonable prior, it is likely very conservative in terms of the parameters generated at the default level: A lower level of transmission, equating to tighter population bottlenecks, would be obtained if we had chosen a more realistic default.

We note that the consequences of error in our default effective viral load are relatively minimal given our consideration of a broad range of effective viral loads. The default describes a single point in a spectrum of exposure levels.

Supplementary Methods 3. Modelling the emission of particles

3.1 Initial conditions

To specify the initial distribution of particles following an emission event we used a system of polar coordinates *{x, θ}*. The initial density of particles in the radial direction was set as a Gaussian distribution with mean position u and standard deviation σ, truncated at radius x=0.

$$\mathrm{a}(x) = \frac{1}{\sigma\sqrt{2\pi}} e^{-\frac{1}{2}\left(\frac{x-\mu}{\sigma}\right)^2} \quad (8)$$

The initial density of particles in the angular direction was also set as a Gaussian distribution, with mean position 0 and standard deviation fixed so that 95% of the distribution was within a specified range $\{-\theta_0, \theta_0\}$, where θ_0 is the spread angle of the emission process. To achieve this we described a model of one-dimensional angular diffusion

$$\mathrm{b}(\theta, t, K) = \frac{1}{\sqrt{4\pi K t}} e^{-(\theta^2/4Kt)} \quad (9)$$

where K is the diffusion coefficient $K(L,\gamma)$, and then solved for t to achieve the desired density

$$t_0(x) = \frac{(x\theta_0/2)^2}{2K} \quad (10)$$

This provides the result

$$b(x,\theta) = \frac{1}{\sqrt{\frac{1}{2}\pi x^2\theta_0^2}} e^{-\frac{1}{2}(\theta^2/x^2\theta_0^2)} \quad (11)$$

The initial distribution (following this first approximation) was then described by the product of these two functions:

$$c(x,\theta) = a(x)b(x,\theta) \quad (12)$$

We set the default angle θ_0 to 45 degrees for coughing and speaking[7,8], and 15 degrees for sneezing[7].

3.2 Speaking model

We modelled the initial distribution of particles from speaking as an instantaneous Gaussian-distributed cloud of particles with zero velocity, at a mean radial distance of 10cm from the infected person, and a standard deviation of 2.5cm. The angular component of the distribution was modelled with spread angle θ_0 of 45 degrees[7].

The distribution of particle sizes emitted from speech was described in a similar way to the distribution for coughing, describing particles with radii $r \in \{1, 2, \ldots, 500\}$ μm, with particles emitted in quantities proportional to a lognormal distribution, with parameters as described in previous literature with u=2.77259 and s=0.597837 [9]. A speaking event was modelled as emitting a volume of 0.579 pL, representing one second of speech: To model continuous speech we modelled 3600 speaking events per hour.

3.3 Sneezing model

We modelled the initial distribution of particles from sneezing as an instantaneous Gaussian-distributed cloud of particles with zero velocity, at a mean distance of 60cm from the infected person, with a standard deviation of 5.5cm. The angular component of the distribution was modelled with spread angle θ_0 of 15 degrees[7].

The sizes of particles emitted from a sneeze were described using a bimodal distribution described by from previous experimental work[10]. As such the proportion of particles of diameter d was set to be proportional to

$$g(d) = \max\{g(a_1, s_1, u_1, d), g(a_2, s_2, u_2, d)\} \qquad (13)$$

where

$$g(a, s, u, d) = \frac{a}{s\sqrt{2\pi}} e^{\left(\frac{-(\log_{10} d - u)^2}{2s^2}\right)} \qquad (14)$$

and the various parameters were given by a_1=4.9359, s_1=0.1479, u_1=2.7334; a_2=2.1649, s_2=0.1483, u_2=2.005. Particle sizes were modelled up to a radius of 1000μm.

A sneeze was modelled as emitting a volume of 7.298 ml, calculated from the distribution of particle sizes and an estimate of 3000 particles being emitted from a sneeze[11]. We modelled emissions of five sneezing events per hour.

Supplementary Methods 4. Analytical solution of the diffusion equation

In the main text we describe the equation

$$\frac{\partial c}{\partial t} = \overbrace{I(x,t)}^{\text{Particle emission}} + \underbrace{K(L,\gamma)\left(\frac{\partial^2 c}{\partial x^2} + \frac{\partial^2 c}{\partial y^2}\right)}_{\text{Turbulent diffusion}} - \overbrace{B(r,c,\gamma)}^{\text{Evacuation}} - \underbrace{S(r,c)}_{\text{Sedimentation}} - \overbrace{D(c)}^{\text{Inactivation}} \quad (15)$$

In our approach we model particle emission as being instantaneous, providing the initial condition of the system at time t=0. We note that $K(L,\gamma)$ is a constant, and that the terms for evacuation, sedimentation, and inactivation can be reduced to a constant pre-factor of c so long as r and γ are constant. We therefore have, in effect:

$$\frac{\partial c}{\partial t} = K\left(\frac{\partial^2 c}{\partial x^2} + \frac{\partial^2 c}{\partial y^2}\right) - \lambda c \quad (16)$$

for some values K and λ, and with an initial condition specified by the model of particle emission. To facilitate the calculation of exposures under a variety of conditions, we generated deterministic solutions to this equation.

4.1 Absorbing boundaries

We first consider the case of absorbing boundary conditions. We suppose that the environment consists of a room with opposite corners at (0,0) and (A,B). We then have the solution

$$c_1(x,y,t) = \left[\sum_{n=1}^{\infty} \sin\left(\frac{n\pi x}{A}\right) \sin\left(\frac{n\pi x_0}{A}\right) e^{-\left(K\left(\frac{n\pi}{A}\right)^2 + \frac{\lambda'}{A^2}\right)t}\right] \text{x} \left[\sum_{m=1}^{\infty} \sin\left(\frac{m\pi x}{B}\right) \sin\left(\frac{m\pi x_0}{B}\right) e^{-\left(K\left(\frac{m\pi}{B}\right)^2 + \frac{\lambda'}{B^2}\right)t}\right] \qquad (17)$$

Where

$$\lambda' = \lambda\left(\frac{A^2B^2}{A^2+B^2}\right) \qquad (18)$$

4.2 Reflecting boundaries

We further consider a case of a room with reflecting boundaries. In this case we consider the function

$$C(x,y,t) = \frac{e^{-\left(\lambda t + \frac{(x-x_0)^2+(y-y_0)^2}{1+4Kt}\right)}}{1+4Kt} \quad (19)$$

This describes a function over an infinite space, without any boundaries. To normalise to an enclosed space with reflecting boundaries we calculate

$$c_2(x,y,t) = \sum_{i=-\infty}^{i=\infty} \sum_{j=-\infty}^{j=\infty} \sum_{k=0}^{k=1} \sum_{l=0}^{l=1} C(2iA + (-1)^k x, 2jB + (-1)^l y, t) \quad (20)$$

for the region $0 \leq x \leq A$ and $0 \leq y \leq B$.

4.3 Initial conditions

Each of our solutions restricts the potential shape of the initial emissions; at time t=0 c_1 has the shape of a two-dimensional Kronecker delta function, while c_2 is a Gaussian. In each case we set a time t_0 at which the shape of the function approximates the shape in which particles are emitted, then simulate diffusion from this point, for example calculating values of $c_1(x,y,t+t_0)$ for times $t \geq 0$.

We carried out the fit between the initial model of the analytical function and the initial conditions using numerical optimisation. For each environment and emissions model studies, we evaluated the initial conditions on a square grid of size 1cm, then calculated t_0 minimising the total of the squared difference between the function and the distribution on this grid.

Supplementary Note 1. Modelling variance in transmission using a gamma-Poisson model

To expand upon our basic model, shown in Figure 1, we expanded the Poisson model used into the original plot to consider a gamma-Poisson model. In short, where in the Poisson model the number of viruses causing infection was given by Poisson distribution with rate *E*, our gamma-Poisson model described a case in which the number of viruses causing infection was given by a Poisson distribution for which the rate was modelled by a Gamma distribution with expectation *E*.

$$G(x) = \frac{e^{-x} x^{E-1}}{\Gamma(E)} \quad (21)$$

A gamma-Poisson model provides a simple way of modelling overdispersion in the exposures received by individuals in an environment. The results obtained were not strongly different from the original model of constant exposure (Supplementary Figure 1). Our physical model of exposure was designed to capture a more realistic distribution of exposure values, and also to account for features such as the non-independence of viruses, as arises from the inclusion of infectious particles in emitted droplets of non-negligible size.

Supplementary Note 2. Alterations and elaborations of our transmission model

Alterations and elaborations of our model produced slightly different outcomes, but essentially the same result in terms of bottleneck sizes. Modelling particle emission via sneezing, we described a case in which a much larger volume of material was emitted, and projected a greater distance from the infected individual. This caused a much broader range

of levels of exposure within a room, with cases of infection caused by exposure to substantially larger particles than either coughing or sneezing (Supplementary Figure 2). In broad scope, the results from this model reflected those obtained from a high-emission cough model: A high effective viral load led to cases in which most bottlenecks were large, and in which values of R_{env} were very high (Supplementary Figure 7).

An elaboration of our model simulating the movement of people made little difference to the bottleneck sizes obtained. By default, all individuals in our model remain stationary for the simulated period. A variant of the nightclub simulation, in which uninfected individuals randomly swapped locations every five minutes, led to a more even distribution of physical exposures (Supplementary Figure 8). However, the proportion of infections that were initiated with tight bottlenecks was close to being unchanged.

Increasing or decreasing ventilation levels for a scenario had a substantial effect on the expected number of people infected, but again made little difference to bottleneck sizes. Keeping other parameters at their default levels, a four-fold increase in ventilation in the office environment was predicted to reduce cases of infection by around 60%, while a four-fold decrease in ventilation was predicted to increase cases by roughly 65% (Supplementary Figure 9). A more detailed consideration of office ventilation has identified comparable results[12]. By c1ontrast, the proportion of bottlenecks of size one varied much less, between 66%, given a four-fold decrease in ventilation, and 79% given a four-fold increase in ventilation. Altering the rate of viral inactivation had a smaller effect on the results than changing ventilation (Supplementary Figure 10).

Altering our model assumptions about how particles interact with walls led to varied outcomes. By default we assumed that walls completely absorbed all particles which contacted them. Relative to this a model in which walls were perfectly reflective increased the overall levels of exposure in each environment, increasing the expected number of cases of infection (Supplementary Figure 11). However, the resulting changes in bottleneck size distributions were more complex: Reflecting boundaries reduced the proportion of infections with small bottlenecks in the lounge, but increased this proportion in the bus environment (Supplementary Figure 12). Our general result remained, with bottlenecks expected to be small in the absence of very high viral load or very high total exposure. An extension to our model in which individuals had some underlying distribution of infectivity levels, modelling greater or lesser amounts of superspreading, produced generally small changes in the bottleneck distributions, with a small increase in the proportion of larger bottlenecks in the lounge given a higher variance in infectivity (Supplementary Figures S13 and S14).

Supplementary Note 3. Modelling variability in infectivity levels

By default our model evaluated statistics over sets of identical individuals, running large numbers of simulations to gather a picture of how a specific set of parameters affected the bottleneck size distribution. We extended this to consider variation in the level of infectivity across a population.

To model variation we added a pre-factor to the exposure values $P_i(r)$. This pre-factor was modelled as following a gamma distribution with parameters $\alpha=a$ and $\beta=a^{-1}$, capturing a variety of potential distributions of host infectivity. We write

$$f(I,a) = \frac{I^{-a-1} a^a e^{-aI}}{\Gamma(a)} \quad (22)$$

In order to simulate a population of infected individuals we generated the centiles of the distribution. Where

$$F(I,a) = \frac{\Gamma(a,0) - \Gamma(a,aI)}{\Gamma(a)} \quad (23)$$

We found values I_j for which $F(I_j,a) \in \{0.001, 0.002, \ldots, 0.999\}$. This gave the modified values

$$P_{i,j}(r) = I_j P_i(r) \quad (24)$$

The values $P_{i,j}$ replaced P_i in equation (17) of the main text, so that we generated

$$n_{i,j,r} = \mathrm{Poisson}\left(P_{i,j}(r)\right) \quad (25)$$

Simulations were repeated over values j to obtain bottlenecks for the mixed population.

To evaluate populations with differing levels of variation between individuals we repeated calculations for values $a \in \{0.1, 0.5, 1, 2, 10\}$.

Supplementary Note 4. On the removal of viruses from the air by inhalation

Within our simulations we assume that the inhalation of viral particles does not affect particle concentration. That is, while the instantaneous exposure of an individual to infectious particles is affected by the local particle concentration, the presence of that individual does not affect the concentration. Further calculations showed that the extent to which viral particles would be removed from the air by inhalation was substantially less than the rate of removal via ventilation.

We assess the proportion of viral particles in a room removed per minute by breathing and by ventilation. As a simplifying assumption, we assume that the viral concentration is uniform throughout the room; our model did not incorporate a more complex reckoning for ventilation.

As such, we modelled the proportion of viral particles removed per minute by breathing as

$$A = \frac{n_e b_e}{v} \quad (26)$$

where n_e is the number of people in the room, be is the number of litres of air inhaled by each person, and v is the volume of the room in litres. For the living room we modelled be equal to 6 litres per minute, equal to a nominal resting rate, while in the nightclub we modelled be equal to 40 litres per minute, corresponding to moderate activity[13]. Other environments had intermediate values of this statistic.

In a similar way we modelled the proportion of viral particles removed per minute by ventilation as

$$p_v = 1 - 0.5^{\gamma/60} \tag{27}$$

where γ is the number of air changes per hour. Here we model ventilation in a simple manner; assuming that an air change is equivalent to feeding in an amount of air equivalent to the volume of the room, thereby reducing the number of viral particles by one half.

Calculating these statistics, we found that in each of our environments the quantity of air removed by ventilation was at least an order of magnitude greater at removing viruses than was removed via breathing (Supplementary Figure 15). Our neglect of the removal of infectious particles via inhalation was therefore of the order of a correction to our ventilation rate.

Supplementary Figures

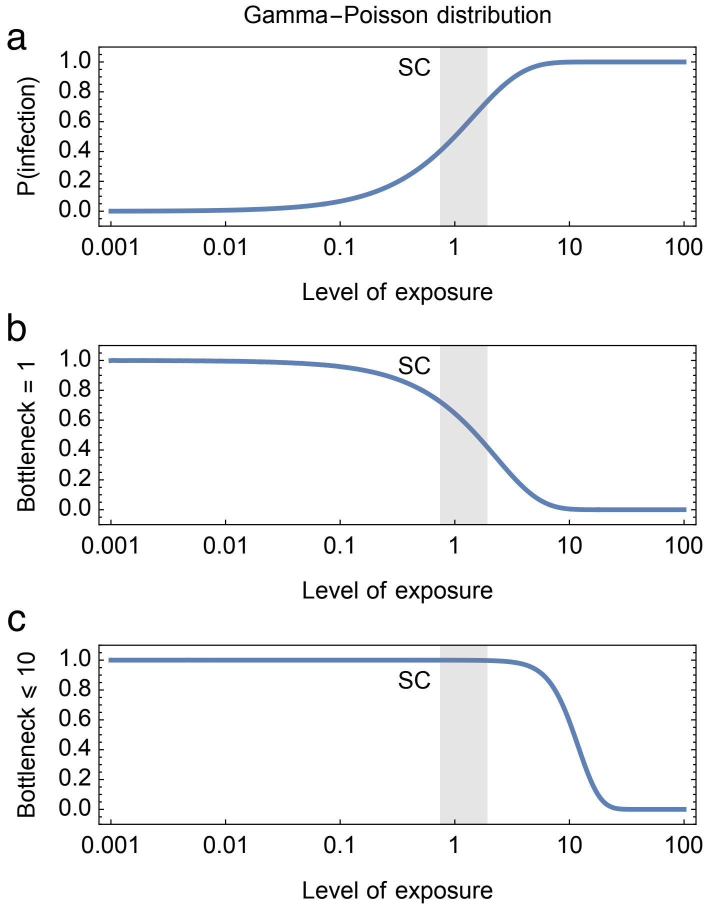

Supplementary Figure 1: Transmission bottleneck estimates under a modified Wells-Riley model of exposure utilising a gamma-Poisson model. In this model the level of exposure describes the expectation of a gamma distribution, from which the number of viruses infecting an individual was drawn via a Poisson distribution. We show **a.** The probability of an individual being infected given the level of exposure. **b.** The proportion of cases of infection in which a single virus initiates infection. **c.** The proportion of cases of infection in which ten or fewer viruses initiate infection. The vertical gray bar provides an estimate of circumstances at the Skagit Choir superspreading event, characterised by the probability of infection. The overdispersion created by the gamma-Poisson model does not have a strong effect upon our results.

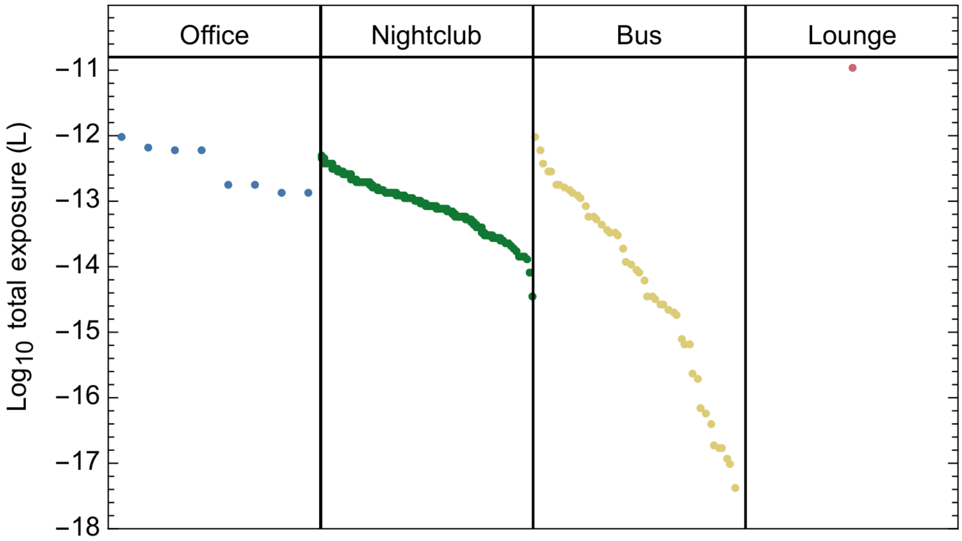

Supplementary Figure 2: Exposure levels generated for individuals in each of the four environments under default parameters. Environments differed in the number of individuals present, and in the shape of the exposure distribution. Distance from the infected person provides one explanation for the differences observed, but the shape of the environment, the extent of ventilation, and the length of time spent in an environment all affect the levels of exposure.

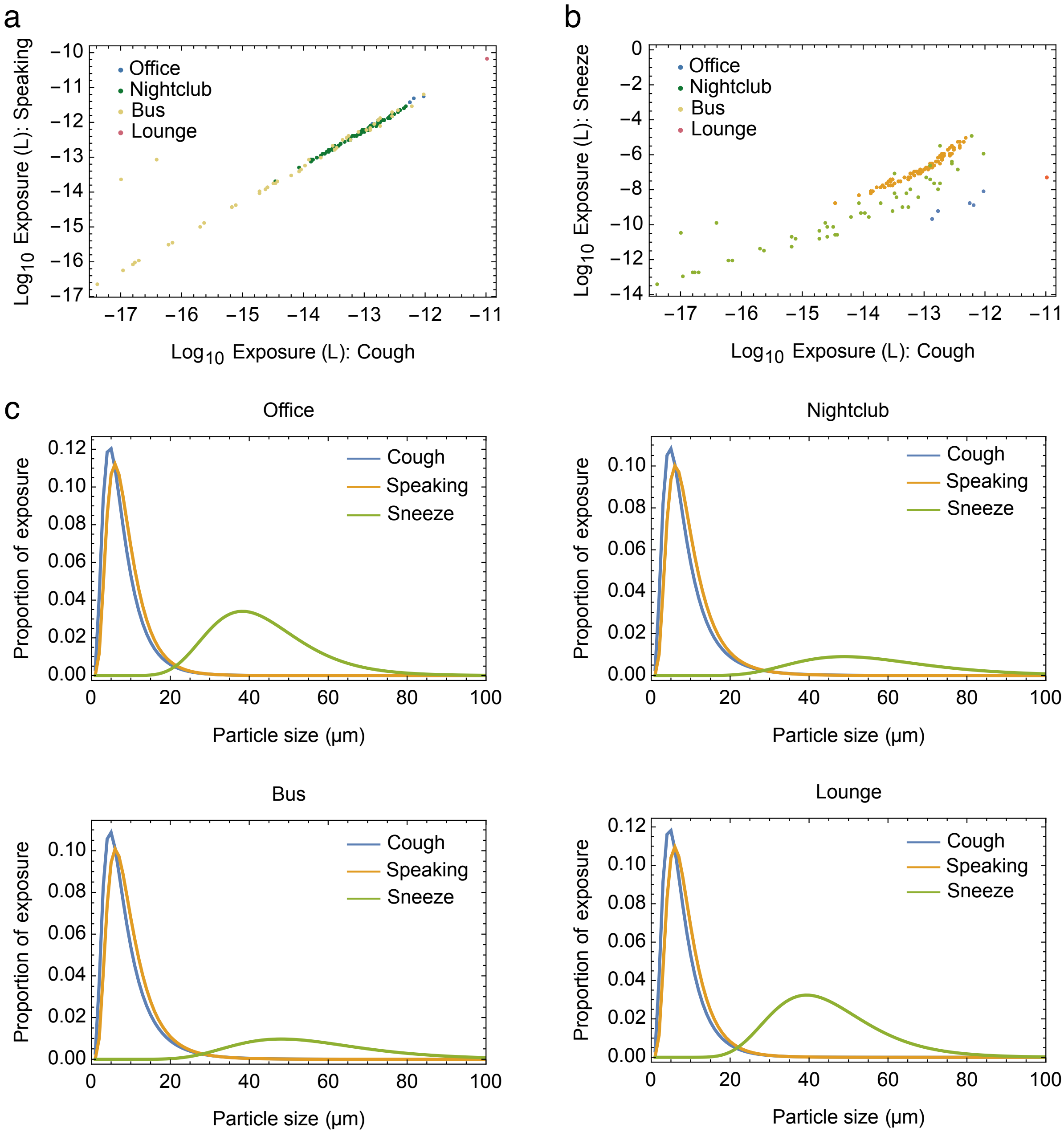

Supplementary Figure 3: Statistics describing exposures for different forms of particle emission, as represented in our model. a. Comparison of exposures for the speech and coughing models across all environments. The magnitude of exposures differ, but are very highly correlated. **b.** Comparison of exposures for the sneezing and coughing models across all environments. **c.** Distribution of particle sizes comprising exposure in each of the different environments.

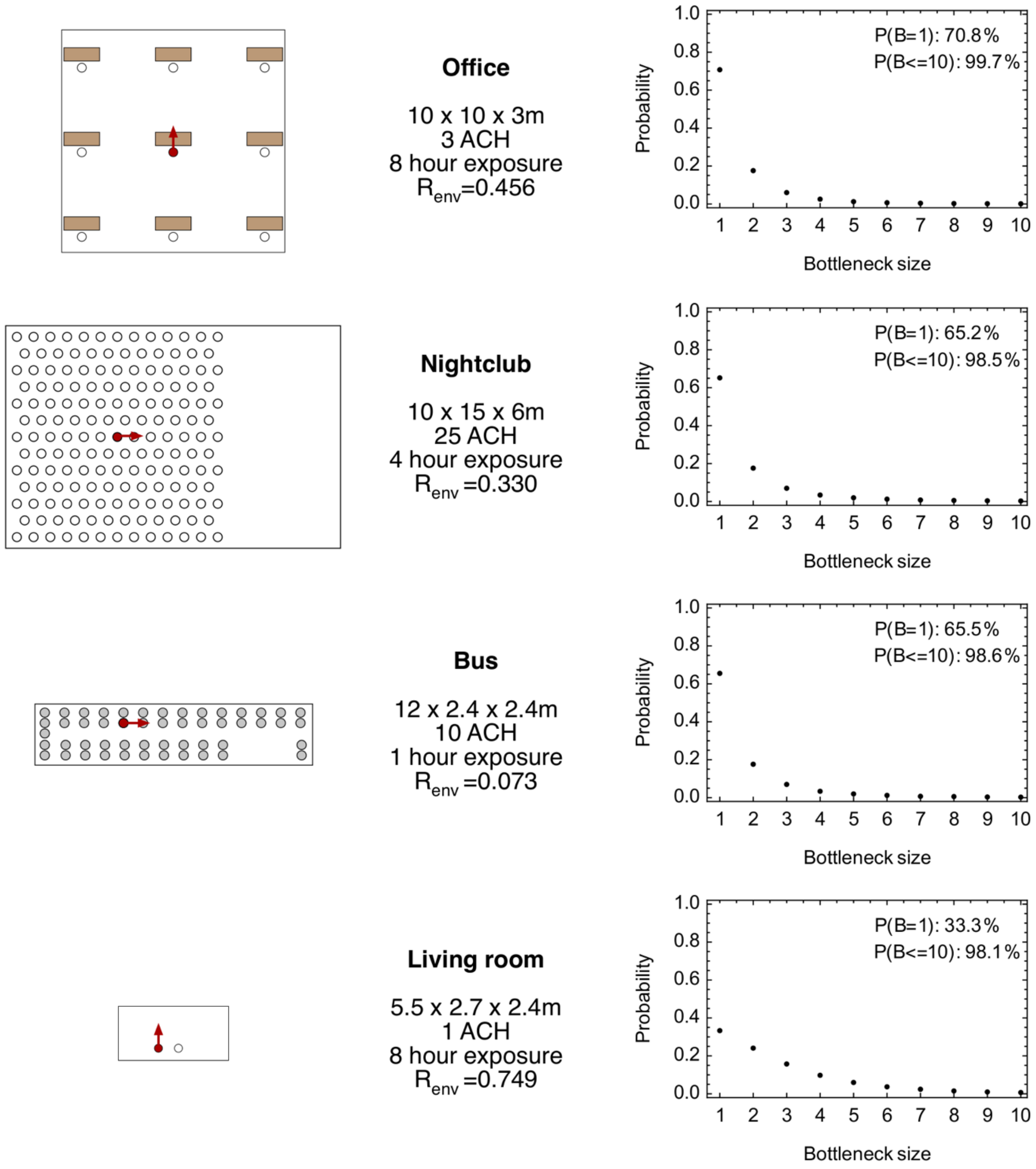

Supplementary Figure 4: Bottleneck size distributions calculated for different scenarios given a model of continuous speech. The value R_{env} describes the expected number of people infected in an environment during the modelled time of exposure. Bottleneck size distributions were calculated from an ensemble of 10^6 simulations generated for each environment.

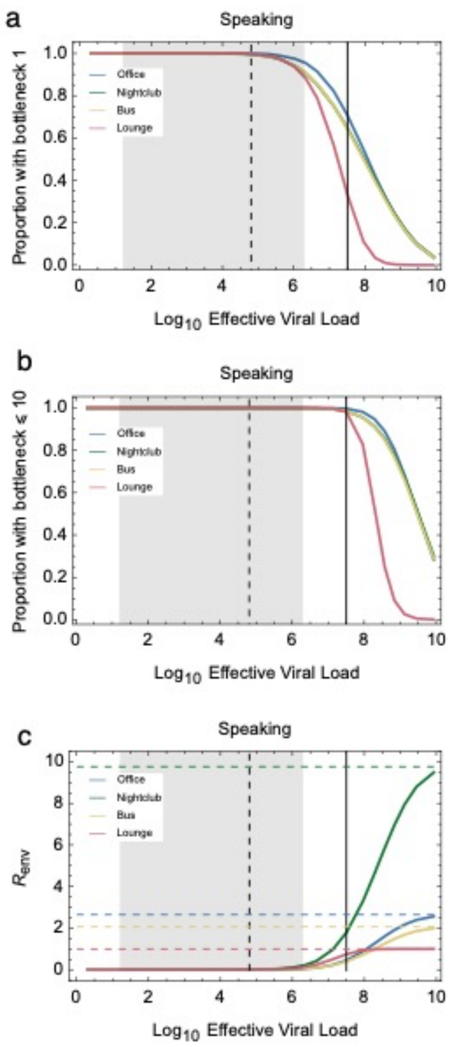

Supplementary Figure 5: Inferred statistics of transmission bottleneck sizes given changes in the effective viral load for a model of continuous speech. Lines connect points calculated at different viral loads for each environment. The dashed vertical black line shows the mean number of plaque forming units at the peak of SARS-CoV-2 infection, while the gray shaded area shows a 95% confidence interval for this statistic. The solid vertical black line shows the effective viral load as specified in the default parameters of our model. Statistics are shown describing **a.** The proportion of transmissions with bottleneck size 1. **b.** The proportion of transmissions with bottleneck size 10 or less. **c.** The expected number of people infected in each environment, R_{env}. Horizontal dashed lines in this figure indicate limit values for each environment, as would occur given a theoretically infinite effective viral load. Results were

calculated from an ensemble of 10^6 simulations generated for each environment and effective viral load.

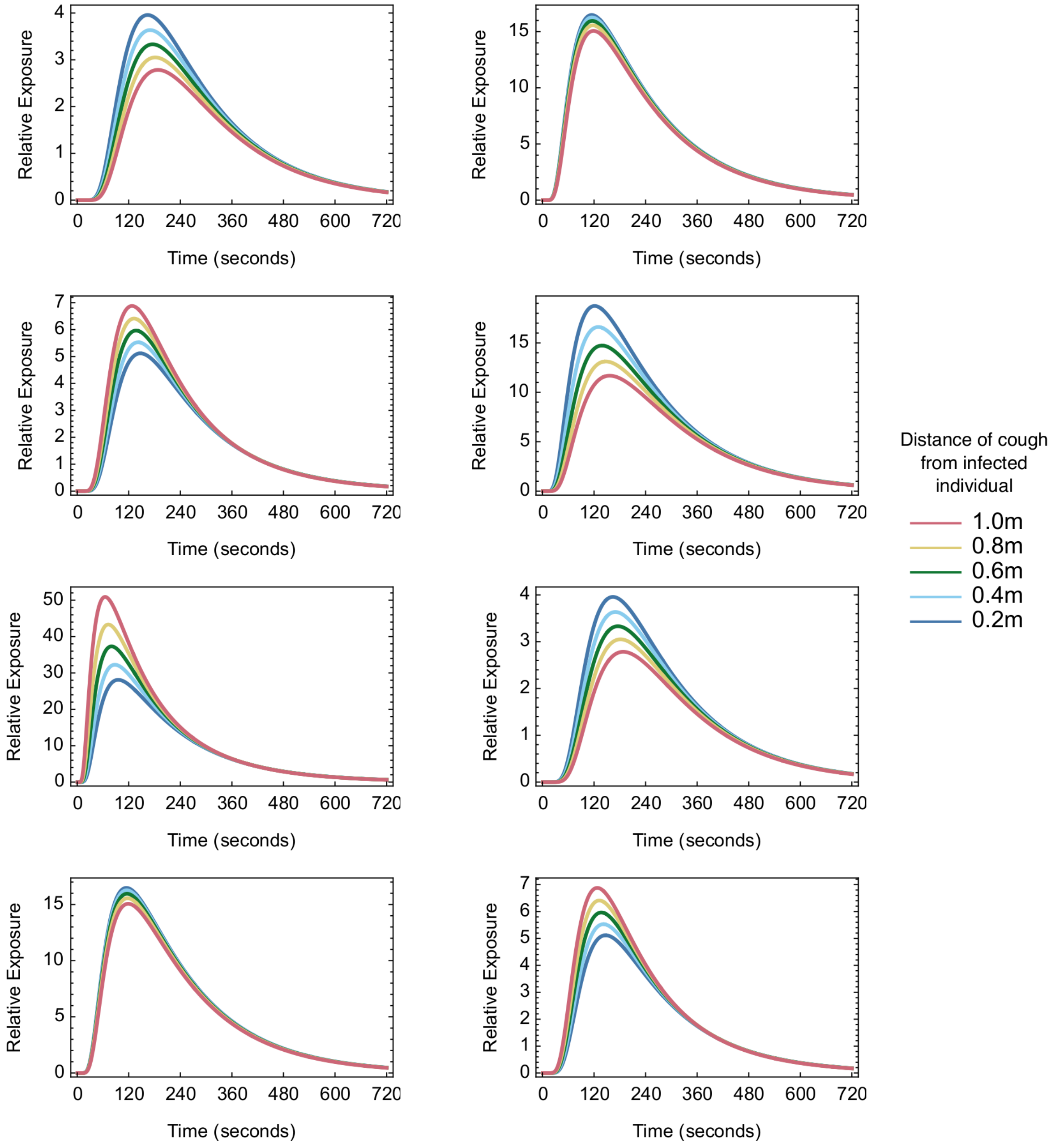

Supplementary Figure 6: Sensitivity analysis to changes in the distance of a cough from the infected individual. Plots show relative exposure levels resulting from a cough for each of the eight uninfected individuals in the office environment. Exposure levels equilibrate after approximately 10 minutes of the one hour simulated.

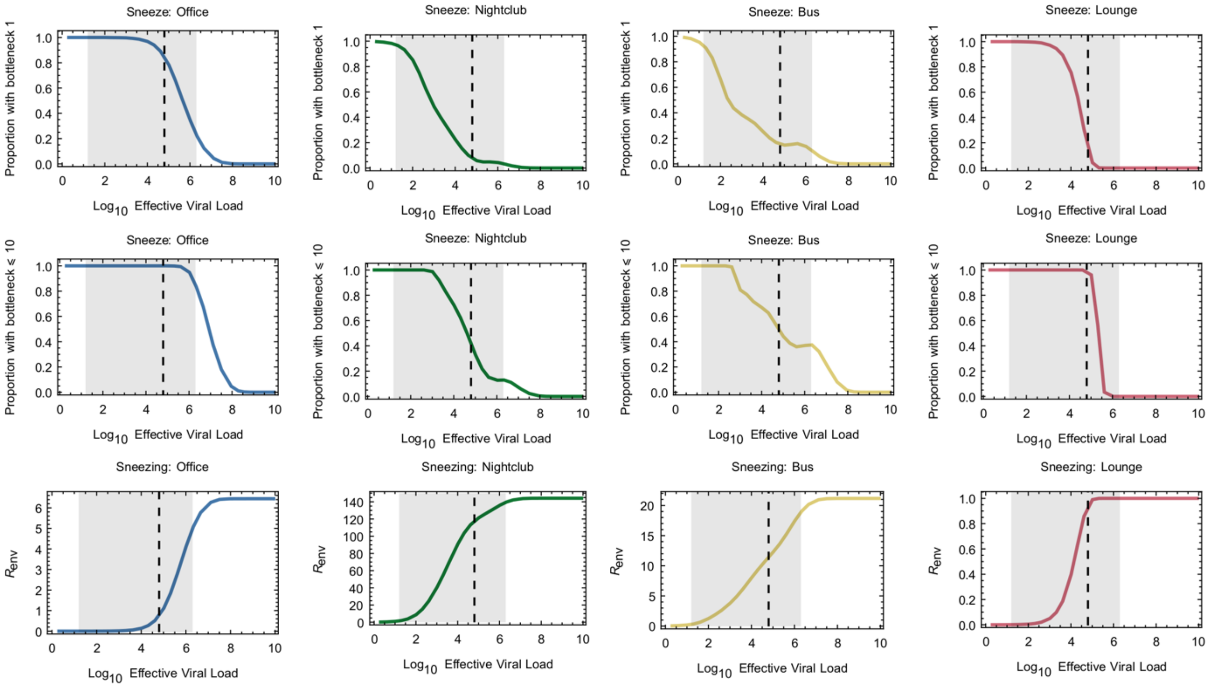

Supplementary Figure 7: Results generated from a model of sneezing. Results show the proportion of transmission events with bottleneck 1, and with bottleneck size less than or equal to 10 for each environment, alongside values of R_{env}, plotted by effective viral load. Results were calculated from an ensemble of 10^6 simulations generated for each environment and effective viral load.

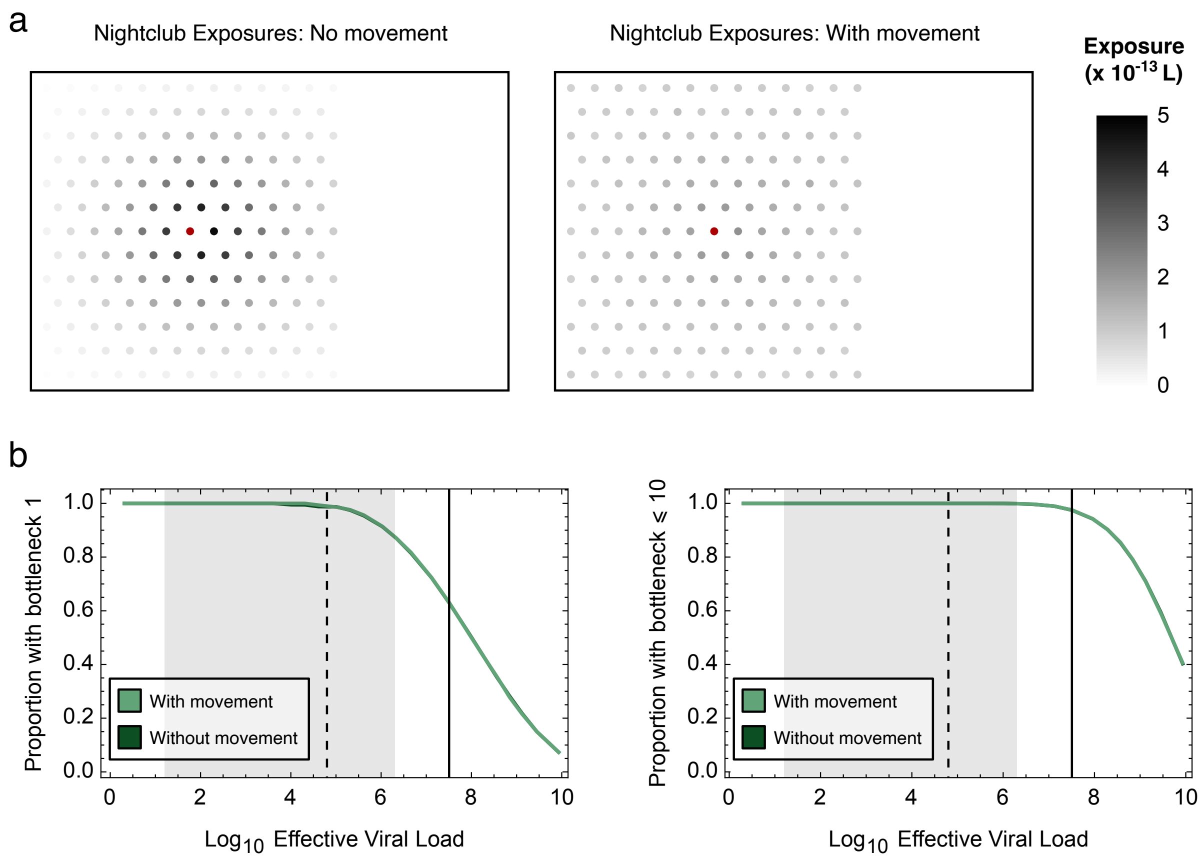

Supplementary Figure 8: Effect of movement on transmission in the nightclub environment. Outputs are shown for the default model, in which uninfected people do not move, and for an alternative model, in which individuals randomly swap locations every five minutes of the four-hour period that was modelled. **a.** Example physical exposures of individuals. Exposures for the movement model are plotted according to the initial positions of individuals in one instantiation of the movement model. **b.** Proportions of transmission events with different sized bottlenecks. Results calculated with and without movement were indistinguishable from one another on the resolution at which data are plotted. Results were calculated from an ensemble of 10^6 simulations generated for each effective viral load.

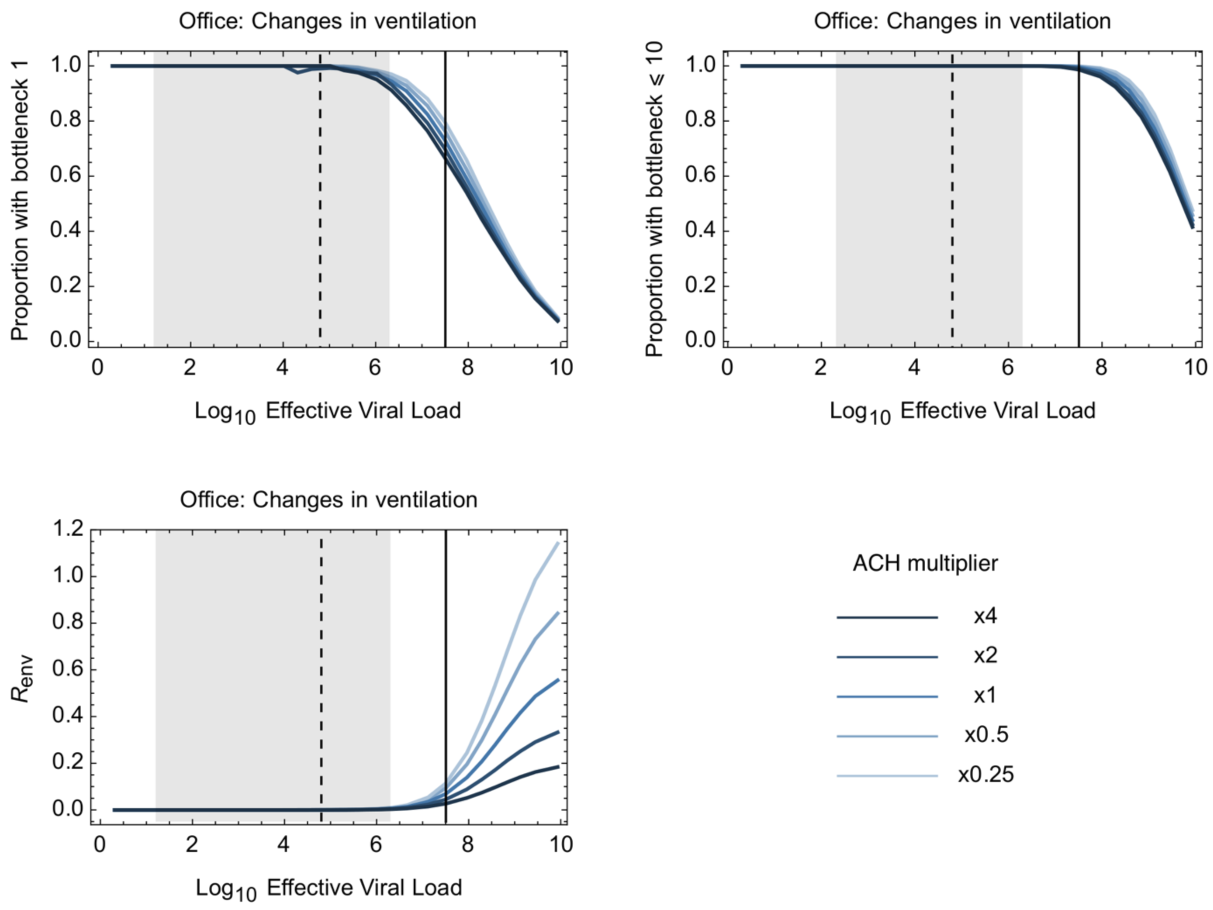

Supplementary Figure 9: Effect of changing ventilation levels on transmission in the office environment. Results plotted in different colours represent outputs of calculations run with different multipliers to the number of air changes per hour in the office, which was set by default as 3. Increased ventilation reduced the number of infections expected to occur in the office. Results were calculated from an ensemble of 10^6 simulations generated for each environment, effective viral load, and ACH multiplier.

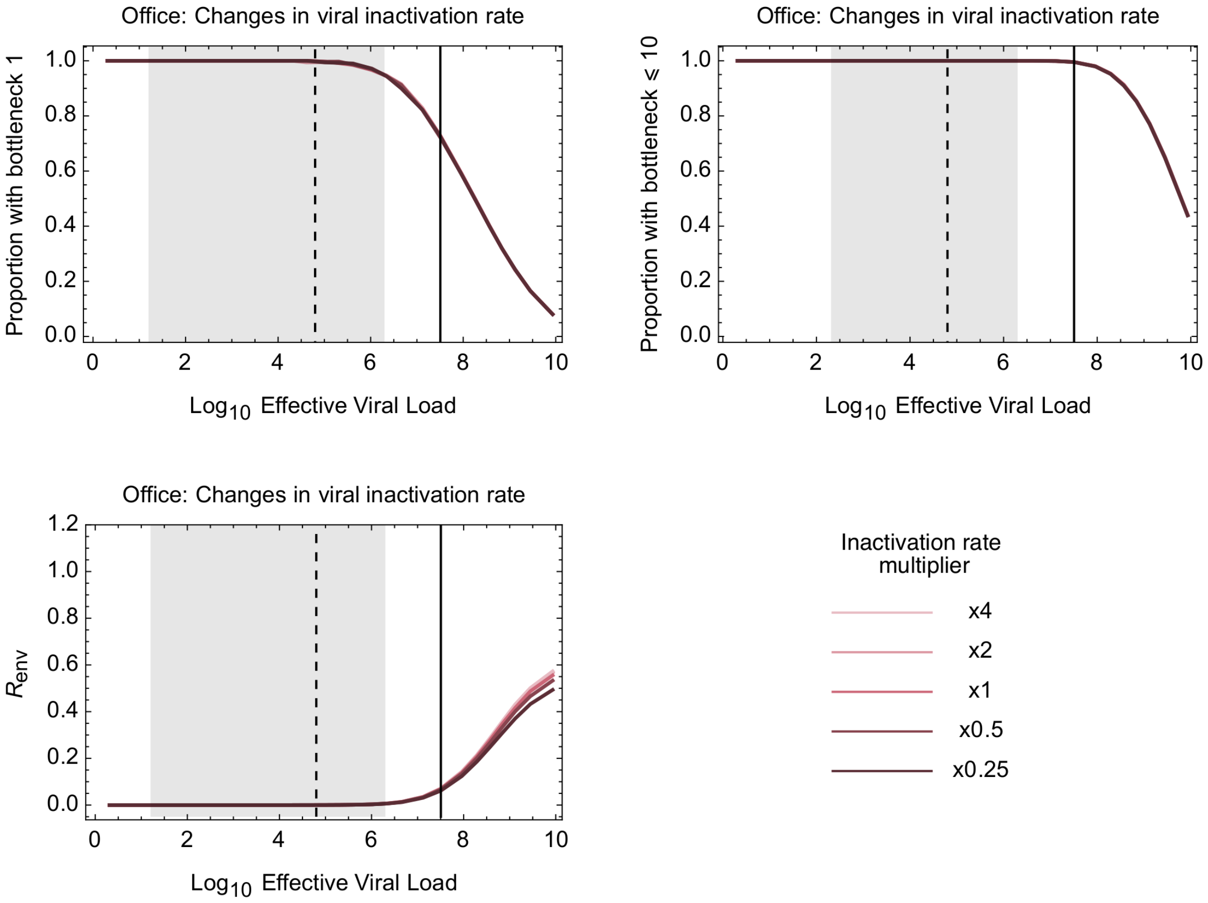

Supplementary Figure 10: Effect of changing the viral inactivation rate on transmission in the office environment. Results plotted in different colours represent outputs of calculations describing the office environment with different multipliers to the viral inactivation rate. An increased inactivation rate led to a small decrease in the number of infections expected to occur. Results were calculated from an ensemble of 10^6 simulations generated for each environment, effective viral load, and inactivation rate multiplier.

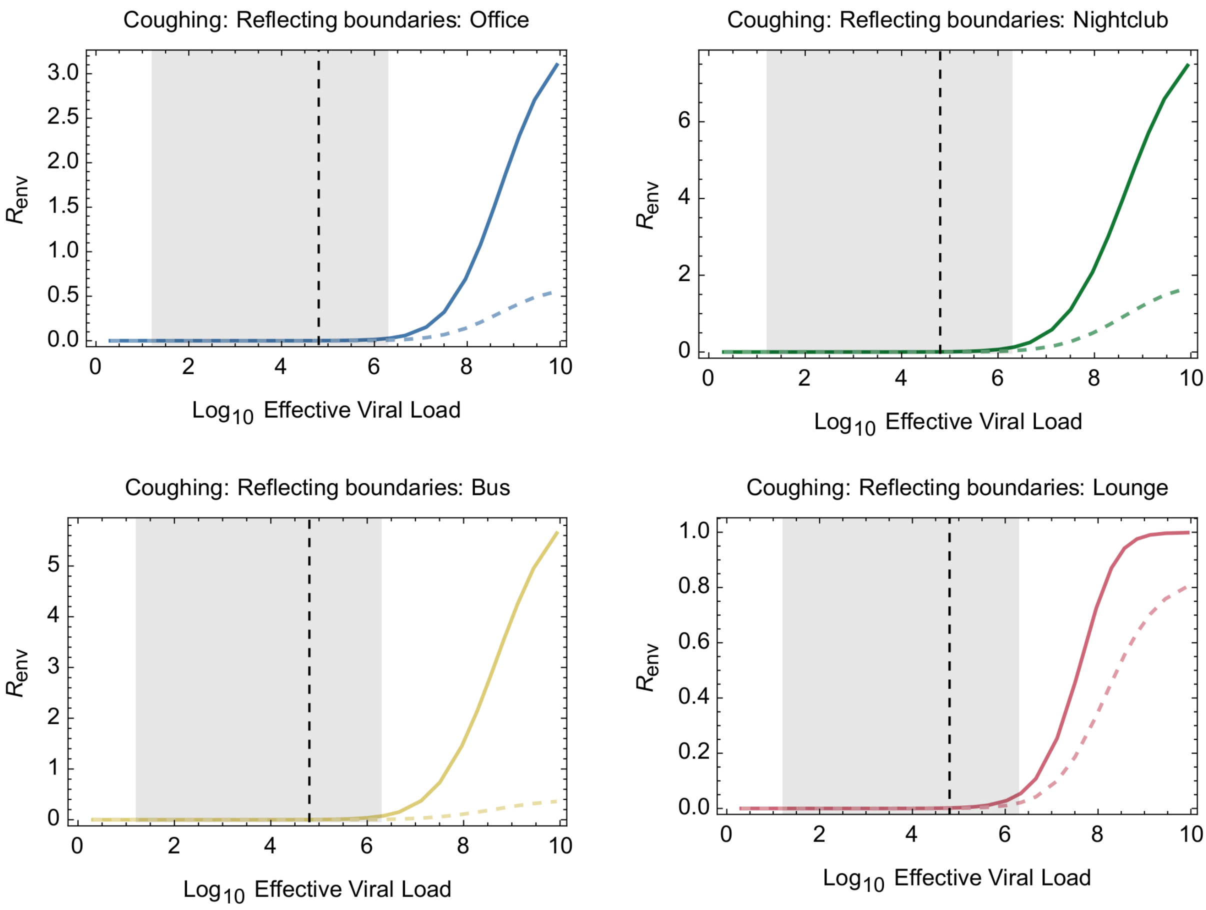

Supplementary Figure 11: Expected numbers of people infected under a model which modelled the reflection of particles at the walls of each environment. Results describe R_{env} for each environment at various effective viral loads. Results are shown in solid lines: Dashed lines represent equivalent results for the default case of absorbing boundaries. Results were calculated from an ensemble of 10^6 simulations generated for each environment and effective viral load.

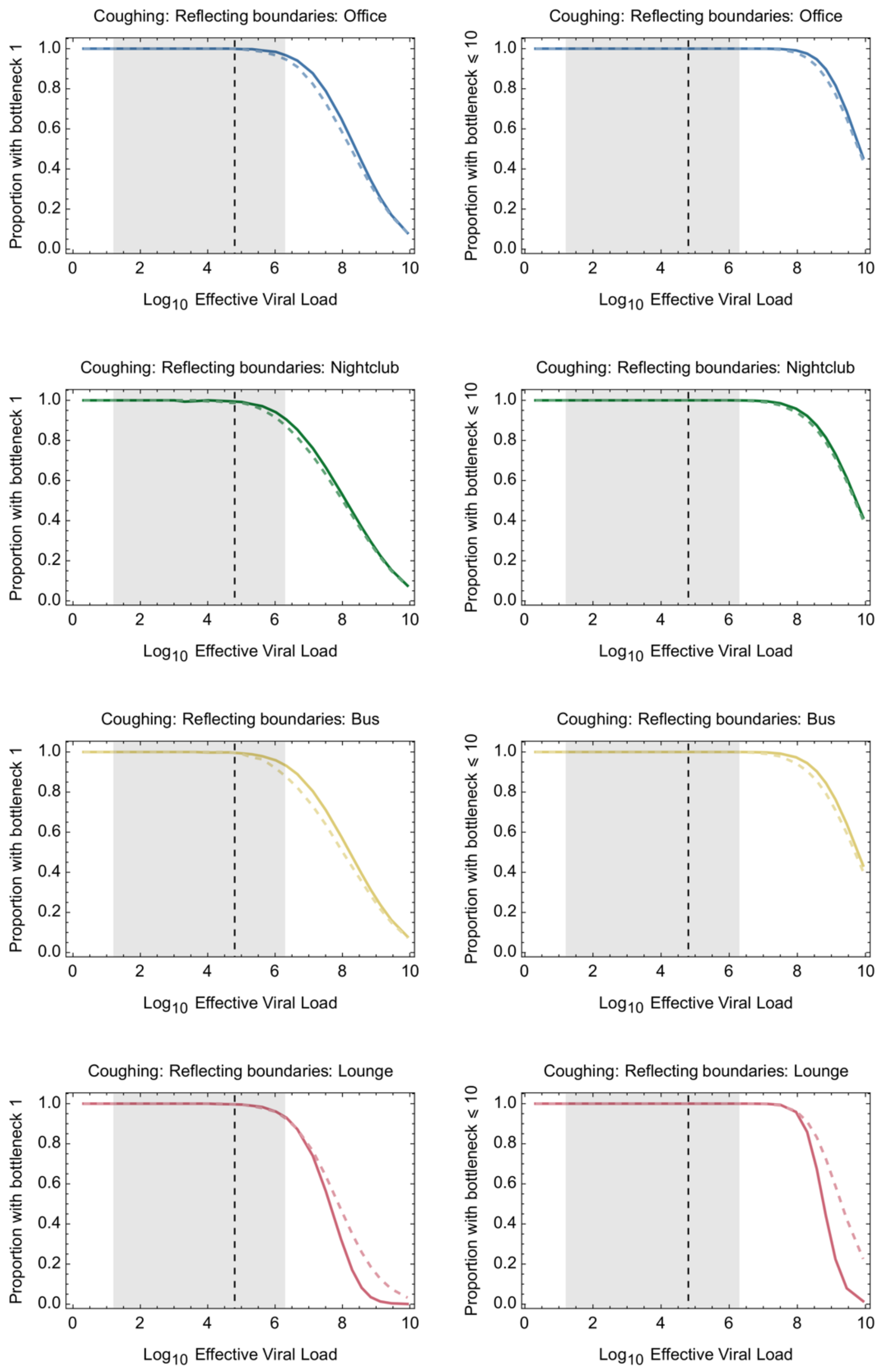

Supplementary Figure 12: Statistics of bottleneck size under a model which modelled the reflection of particles at the walls of each environment. Results are shown in solid lines: Dashed lines represent equivalent results for the default case of absorbing boundaries. Results were calculated from an ensemble of 10^6 simulations generated for each environment and effective viral load.

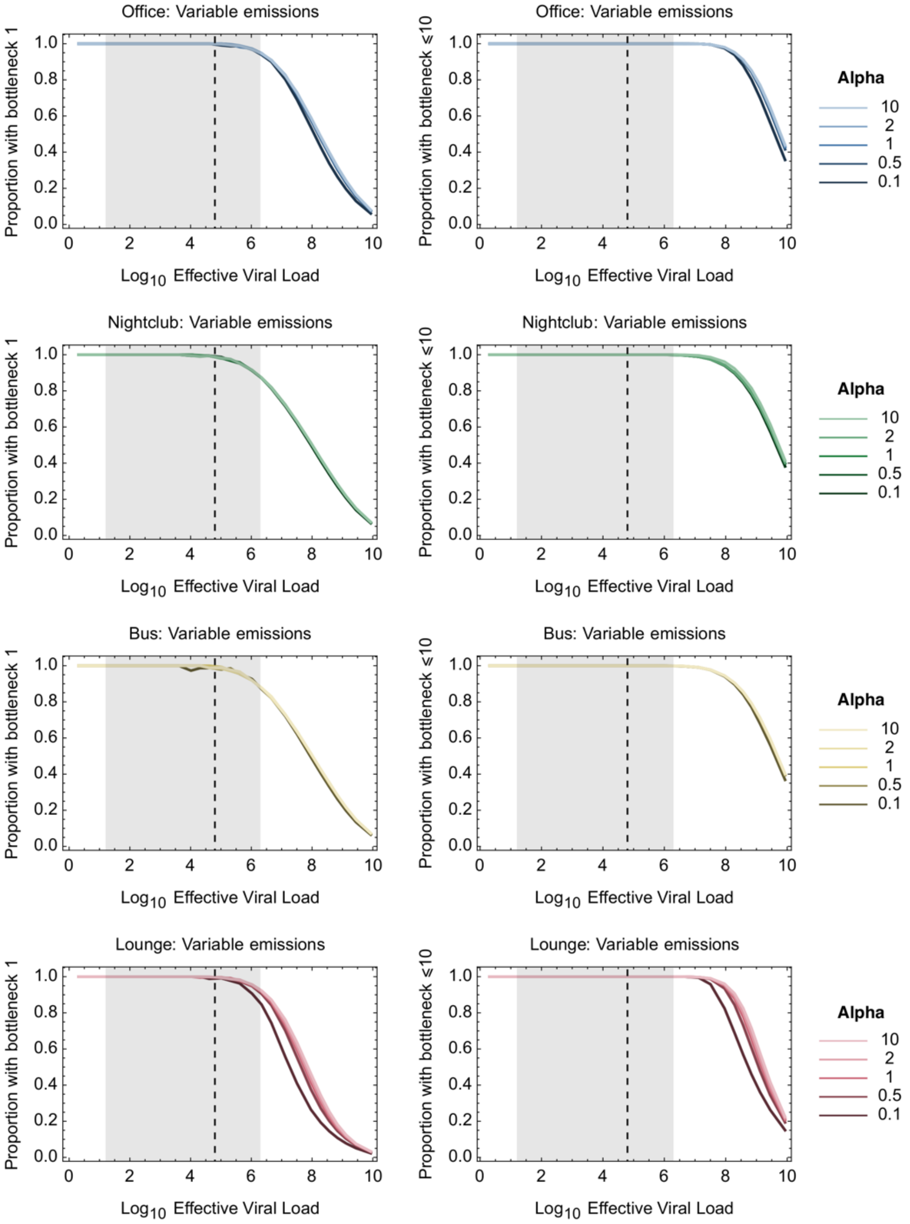

Supplementary Figure 13: Statistics of bottleneck size under a model in which the infected individual had a distribution of infectivity values. Infectivity values were characterised according to a Gamma distribution with parameters α and $1/\alpha$, having mean 1. A low value of α here corresponds to a greater diversity of infectivity values. Results were calculated from an ensemble of 10^6 simulations generated for each environment, effective viral load, and value of α.

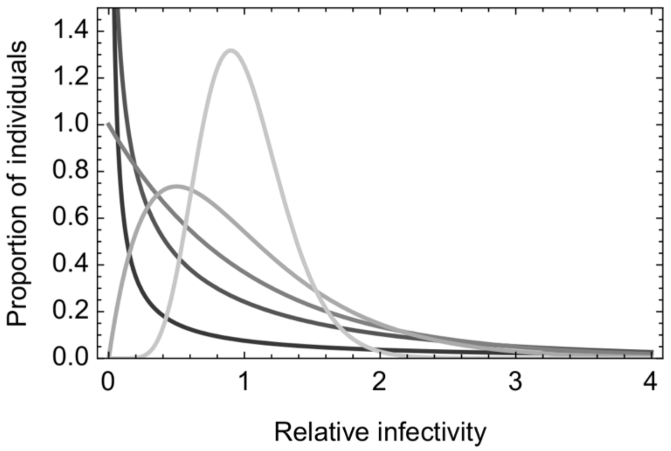

Supplementary Figure 14: Infectivity distributions used in our model. Infectivity values were characterised according to a Gamma distribution with parameters α and $1/\alpha$, each distribution having mean value 1.

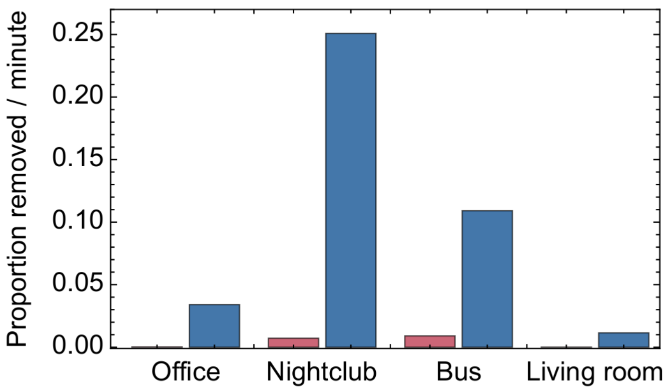

Supplementary Figure 15: Proportion of viruses removed from the environment by breathing and by ventilation. Values are shown as the fraction of the total volume removed per minute, calculated from the model parameters.

Supplementary References

1. He, X. *et al.* Temporal dynamics in viral shedding and transmissibility of COVID-19. *Nat Med* **26**, 672–675 (2020).
2. Park, M., Cook, A. R., Lim, J. T., Sun, Y. & Dickens, B. L. A Systematic Review of COVID-19 Epidemiology Based on Current Evidence. *JCM* **9**, 967 (2020).
3. Ng, O. T. *et al.* SARS-CoV-2 seroprevalence and transmission risk factors among high-risk close contacts: a retrospective cohort study. *The Lancet Infectious Diseases* **21**, 333–343 (2021).
4. Chen, Y.-H. *et al.* Excess mortality associated with the COVID-19 pandemic among Californians 18–65 years of age, by occupational sector and occupation: March through November 2020. *PLoS ONE* **16**, e0252454 (2021).

5. Hakki, S. *et al.* Onset and window of SARS-CoV-2 infectiousness and temporal correlation with symptom onset: a prospective, longitudinal, community cohort study. *The Lancet Respiratory Medicine* **10**, 1061–1073 (2022).

6. Ferretti, L. *et al.* Digital measurement of SARS-CoV-2 transmission risk from 7 million contacts. *Nature* (2023) doi:10.1038/s41586-023-06952-2.

7. Han, M. *et al.* Experimental measurements of airflow features and velocity distribution exhaled from sneeze and speech using particle image velocimetry. *Building and Environment* **205**, 108293 (2021).

8. Li, M. *et al.* Towards realistic simulations of human cough: Effect of droplet emission duration and spread angle. *International Journal of Multiphase Flow* **147**, 103883 (2022).

9. Wang, Y., Xu, G. & Huang, Y.-W. Modeling the load of SARS-CoV-2 virus in human expelled particles during coughing and speaking. *PLoS ONE* **15**, e0241539 (2020).

10. Han, Z. Y., Weng, W. G. & Huang, Q. Y. Characterizations of particle size distribution of the droplets exhaled by sneeze. *J. R. Soc. Interface.* **10**, 20130560 (2013).

11. Dhand, R. & Li, J. Coughs and Sneezes: Their Role in Transmission of Respiratory Viral Infections, Including SARS-CoV-2. *Am J Respir Crit Care Med* **202**, 651–659 (2020).

12. Obeid, S. *et al.* Airborne respiratory aerosol transport and deposition in a two-person office using a novel diffusion-based numerical model. *J Expo Sci Environ Epidemiol* (2023) doi:10.1038/s41370-023-00546-w.

13. Pleil, J. D., Ariel Geer Wallace, M., Davis, M. D. & Matty, C. M. The physics of human breathing: flow, timing, volume, and pressure parameters for normal, on-demand, and ventilator respiration. *J. Breath Res.* **15**, 042002 (2021).
