# Supplementary material for: The airborne transmission of viruses causes tight transmission bottlenecks
Source: Nat Commun. 2024 Apr 26;15:3540. doi: 10.1038/s41467-024-47923-z (PMC11053022; doi:10.1038/s41467-024-47923-z)
Supplement: Supplementary file 3 — Reporting Summary [file 41467_2024_47923_MOESM3_ESM.pdf]

nature portfolio

Corresponding author(s): *Double-anonymous peer review submissions: write DAPR and your manuscript number here instead of author names.*

Last updated by author(s): *YYYY-MM-DD*

Reporting Summary

Nature Portfolio wishes to improve the reproducibility of the work that we publish. This form provides structure for consistency and transparency in reporting. For further information on Nature Portfolio policies, see our Editorial Policies and the Editorial Policy Checklist.

Statistics

For all statistical analyses, confirm that the following items are present in the figure legend, table legend, main text, or Methods section.

n/a	Confirmed	
☐	☒	The exact sample size (*n*) for each experimental group/condition, given as a discrete number and unit of measurement
☐	☒	A statement on whether measurements were taken from distinct samples or whether the same sample was measured repeatedly
☒	☐	The statistical test(s) used AND whether they are one- or two-sided<br>*Only common tests should be described solely by name; describe more complex techniques in the Methods section.*
☐	☒	A description of all covariates tested
☐	☒	A description of any assumptions or corrections, such as tests of normality and adjustment for multiple comparisons
☐	☒	A full description of the statistical parameters including central tendency (e.g. means) or other basic estimates (e.g. regression coefficient) AND variation (e.g. standard deviation) or associated estimates of uncertainty (e.g. confidence intervals)
☒	☐	For null hypothesis testing, the test statistic (e.g. *F*, *t*, *r*) with confidence intervals, effect sizes, degrees of freedom and *P* value noted<br>*Give P values as exact values whenever suitable.*
☒	☐	For Bayesian analysis, information on the choice of priors and Markov chain Monte Carlo settings
☒	☐	For hierarchical and complex designs, identification of the appropriate level for tests and full reporting of outcomes
☒	☐	Estimates of effect sizes (e.g. Cohen's *d*, Pearson's *r*), indicating how they were calculated

Our web collection on statistics for biologists contains articles on many of the points above.

Software and code

Policy information about availability of computer code

Data collection	Data describing levels of exposure were generated using computational simulation methods. The code to carry out this generation is available here: https://github.com/cjri/DiffusionCode. Scripts are provided to enable a third party to directly interact with the code and regenerate results.
Data analysis	The conversion of levels of simulated levels of physical exposure into transmission bottleneck sizes was conducted using computer code. The code to carry out this analysis is available here: https://github.com/cjri/DiffusionCode.

For manuscripts utilizing custom algorithms or software that are central to the research but not yet described in published literature, software must be made available to editors and reviewers. We strongly encourage code deposition in a community repository (e.g. GitHub). See the Nature Portfolio guidelines for submitting code & software for further information.

Data

Policy information about availability of data

All manuscripts must include a data availability statement. This statement should provide the following information, where applicable:

- Accession codes, unique identifiers, or web links for publicly available datasets
- A description of any restrictions on data availability
- For clinical datasets or third party data, please ensure that the statement adheres to our policy

Data underlying the figures in our manuscript has been included in a GitHub repository here: https://github.com/cjri/DiffusionCodeData.

Research involving human participants, their data, or biological material

Policy information about studies with human participants or human data. See also policy information about sex, gender (identity/presentation), and sexual orientation and race, ethnicity and racism.

Reporting on sex and gender	N/A
Reporting on race, ethnicity, or other socially relevant groupings	N/A
Population characteristics	N/A
Recruitment	N/A
Ethics oversight	N/A

Note that full information on the approval of the study protocol must also be provided in the manuscript.

Field-specific reporting

Please select the one below that is the best fit for your research. If you are not sure, read the appropriate sections before making your selection.

☐ Life sciences ☐ Behavioural & social sciences ☒ Ecological, evolutionary & environmental sciences

For a reference copy of the document with all sections, see nature.com/documents/nr-reporting-summary-flat.pdf

Ecological, evolutionary & environmental sciences study design

All studies must disclose on these points even when the disclosure is negative.

Study description	We use simulation methods to describe the physical process of respiratory viral transmission with the aim of studying the number of viruses initiating cases of infection. A simulation describes the physical forces affecting the spread of particles emitted by an infected person within a given environment, and the consequent levels of exposure of previously uninfected individuals in that environment. Exposures were then converted into stochastic outcomes of a process of people being infected with a number of viruses (possibly zero). A total of 10^6 simulations were generated for each of a range of conditions, describing different environments and patterns of particle emission. Statistics describing transmission bottleneck sizes were generated across ensembles of simulations.
Research sample	We describe a broad range of patterns of particle emissions, broadly modelling coughing, speaking, and sneezing, with modifications to these patterns, for example arbitrarily increasing the volume of particles emitted. We model different environments of different sizes, with different levels of occupation, and with different environmental parameters, considering a broad range of high or low ventilation rates.
Sampling strategy	Our study considers a set of possible outcomes of the stochastic process of respiratory viral transmission. In this process individuals may or may not be infected following a period of proximity to an infected person. Our sampling strategy was to generate a large number of simulations, each simulation describing one outcome from the process in question. We carried out 10^6 simulations of each environment and set of parameters.
Data collection	Our simulation code generates files which were stored on a computer for further analysis. Chris Illingworth and Patrick Sinclair wrote code. Chris Illingworth ran the final set of simulations to generate the data used in this publication.
Timing and spatial scale	In simulations, data was collected from the instant that an emission was made, with the level of physical exposure being measured every second for a period of one hour after the emission. An interval of one second was chosen for reasons of feasibility. A cutoff of one hour was chosen as after this period the level of physical exposure had dropped to several orders of magnitude below its peak value.
Data exclusions	We didn't exclude any data.
Reproducibility	In so far as the data are generated using computational code, it is possible to regenerate all of the data from the code. We have provided the code for this purpose.
Randomization	This is not really a relevant question for the purpose of our study.
Blinding	Our study does not involve e.g. a placebo treatment versus a trial. We are looking at a basic phenomenon, rather than a difference between two treatments or approaches.

Did the study involve field work? ☐ Yes ☒ No

Reporting for specific materials, systems and methods

We require information from authors about some types of materials, experimental systems and methods used in many studies. Here, indicate whether each material, system or method listed is relevant to your study. If you are not sure if a list item applies to your research, read the appropriate section before selecting a response.

Materials & experimental systems

n/a	Involved in the study
☒	☐ Antibodies
☒	☐ Eukaryotic cell lines
☒	☐ Palaeontology and archaeology
☒	☐ Animals and other organisms
☒	☐ Clinical data
☒	☐ Dual use research of concern
☒	☐ Plants

Methods

n/a	Involved in the study
☒	☐ ChIP-seq
☒	☐ Flow cytometry
☒	☐ MRI-based neuroimaging

Plants

Seed stocks	N/A
Novel plant genotypes	N/A
Authentication	N/A
